# Clinical and Prognostic Relevance of B7-H3 and Indicators of Glucose Metabolism in Colorectal Cancer

**DOI:** 10.3389/fonc.2020.546110

**Published:** 2020-09-15

**Authors:** Ting Zhang, Yufen Jin, Xin Jiang, Longhai Li, Xiaowei Qi, Yong Mao, Dong Hua

**Affiliations:** ^1^Institue of Cancer, Affiliated Hospital of Jiangnan University, Wuxi, China; ^2^Wuxi School of Medicine, Jiangnan University, Wuxi, China; ^3^Department of Pathology, Affiliated Hospital of Jiangnan University, Wuxi, China; ^4^Department of Oncology, Affiliated Hospital of Jiangnan University, Wuxi, China

**Keywords:** B7-H3, glucose metabolism, colorectal cancer, correlation, prognosis

## Abstract

**Objective:**

This study aimed to investigate the clinical and prognostic relevance of B7-H3 expression and indicators of glucose metabolism in patients with colorectal cancer (CRC).

**Methods:**

Using immunohistochemistry, the expression of B7-H3 was detected in a total of 213 formalin-fixed paraffin-embedded CRC tissue specimens. Furthermore, levels of fasting blood glucose (FBG), lactic dehydrogenase (LDH), and fructosamine (FMN) as indicators of glucose metabolism were analyzed in CRC patients and stratified into high or low expression sub-groups based on Youden Index. The relationship between B7-H3, FBG, LDH, FMN expression, and clinicopathological characteristics were also evaluated to establish their prognostic significance in patients with CRC.

**Results:**

B7-H3 was highly expressed in CRC tissue. The positive rates of B7-H3 expression was 63.8% (136/213). We found a linear correlation between B7-H3 and FBG in depth of tumor invasion (T3/4) (*p* = 0.037, *r* = 0.259), lymph node metastasis (N0) (*p* = 0.004, *r* = 0.259), and TNM stage (I/II) (*p* = 0.009, *r* = 0.242). High expression of FBG, LDH, FMN [hazard ratio (HR) = 1.916, 95% CI: 1.223–3.00, *p* = 0.005; HR = 1.801, 95% CI: 1.153–2.813, *p* = 0.010; HR = 2.154, 95% CI: 1.336–3.472, *p* = 0.002], respectively, was identified as a significant independent predictor of poor overall survival (OS). Although B7-H3 expression did not affect OS, CRC patients expressing both high B7-H3 and high FMN contributed to a significant decrease in OS (HR = 1.881, 95%CI: 1.059–3.339, *p* = 0.031). Moreover, with low expression of B7-H3, high expression of FBG, LDH and FMN were also recognized as predictors of inferior OS (HR = 3.393, 95% CI: 1.493-7.709, *p* = 0.004; HR = 7.107, 95% CI: 2.785–18.138, *p* = 0.000; HR = 2.800, 95% CI: 1.184–6.625, *p* = 0.019).

**Conclusion:**

B7-H3 combined with FBG, LDH, or FMN, could reflect the clinical outcomes of patients with CRC.

## Introduction

Colorectal cancer (CRC) remains the third most frequently diagnosed gastrointestinal tract malignancy in men and second in women, globally. Although the mortality rate of CRC has been declining progressively, it still represents the third leading cause of cancer-associated mortality for both men and women (1). In contrast to these declines, CRC remains the most rapidly increasing cancer in China over the past few decades. This rise in CRC incidence has become one of the major public health concerns causing a substantial health burden to families and also contributing to overburdened healthcare systems (2). Recently, Gu et al. summarized tobacco smoking, alcohol abuse, obesity, low physical activity, low fruit and fiber consumption, and high intake of red and processed meat as the significant attributable causes of CRC in China (3). Moreover, these risk factors were also associated with poor prognosis of CRC, particularly in CRC patients with diabetes mellitus (DM).

In the past three decades, the prevalence of DM and associated health complications has substantially increased in the world, and DM represented the ninth leading cause of death (4). Notably, China has rapidly emerged as a region with DM global epidemic, owing to an unhealthy diet and a sedentary lifestyle as critical drivers (5). Moreover, accumulating epidemiological studies have indicated a positive association of DM with the risk of CRC (6). Several similar risk factors, including western lifestyle, have been reported between DM and CRC. Furthermore, fasting blood glucose (FBG), as an indicator of DM, was also found to be significantly related to the risk of CRC (7). Also, aerobic glycolysis is the most common energy metabolism characteristic of cancer cells. Previously, Graziano et al. analyzed mRNA expression of the key enzymes including GLUT1, LDHA, HK1, PKM2, and VDAC1 mRNA involved in glycolysis in CRC patients and revealed that expression levels were significantly higher in primary tumor tissues compared with normal mucosa (8). Besides, altered expression of genes involved in glucose uptake and glycolysis was also reported in CRC tissues (9, 10).

Immune checkpoint protein B7 homolog 3 (B7-H3 or CD276), a type I transmembrane glycoprotein, belongs to the B7 and CD28 superfamilies. While limited-expression has been observed in normal healthy tissues, overexpression of B7-H3 has been reported in a variety of malignancy and plays a crucial role in tumor progression (11). Clinically, B7-H3 overexpression in tumors has been associated with poor clinical outcomes (12). Moreover, increasing studies have indicated that the aberrant expression of B7-H3 is a consistent characteristic of CRC. Thus, B7-H3 detection might be an effective means to predict the prognosis in patients with CRC. Besides, Fang et al. demonstrated an increased level of soluble B7-H3 in type 1 diabetes patients compared with healthy controls (13), implying that B7-H3 might be a promising biomarker in the pathogenesis of diabetes. Furthermore, by inducing expression of the key glycolytic enzyme, hexokinase 2 (HK2), overexpression of B7-H3 has been documented to increase the rate of glucose consumption and lactate production (14). These findings indicated that B7-H3 might be a novel regulator of glucose metabolism in CRC cells and a promising therapeutic target for CRC. Moreover, in hypoxic conditions, lactic dehydrogenase (LDH) has been known to convert pyruvate to lactic acid to support tumor cells, and elevated serum LDH has been reported to express in cancer and considered to be an independent prognostic predictor (15, 16). Besides, fructosamine (FMN) predominantly represents a measure of glycated albumin, which is the most abundant of the serum proteins. As the half-life of albumin is shorter than that of hemoglobin A1c (HbA1c), FMN reflects a shorter duration of glycemic control over the past 2–3 weeks period. This evidence indicates that FMN could be used as a short-term marker of glucose control. Moreover, elevated FMN levels have been associated with an increased risk of colorectal adenoma, a precursor of CRC (17).

Despite these pieces of evidence, only limited studies have been focused on B7-H3 and the indicators of glucose metabolism (FBG, LDH, and FMN) in CRC patients. Therefore, the present study was initiated to investigate the clinical and prognostic significance of B7-H3, FBG, LDH, and FMN in patients CRC.

## Materials and Methods

### Patients and Samples Collection

The medical records of patients who received a histopathological diagnosis and underwent surgical resection for CRC at the Affiliated Hospital of Jiangnan University between June 2008 and December 2011 were included. A total of 213 formalin-fixed paraffin-embedded CRC tissue samples were included in this study. For this study, only histopathologically confirmed cases were included. Besides, patients who received chemo- or radiotherapy before surgery and cases with incomplete clinical data were excluded from the study. For all patients, pathological stages were defined according to the seventh edition American Joint Committee on Cancer (AJCC) cancer staging manual using available clinical and pathologic tumor, node, and metastasis data. Patient demographic data, including age and gender and clinicopathologic characteristics, such as tumor size and histologic grade and FBG, LDH, and FMN from clinical laboratory investigation, were also collected. This study was approved by the Medical Ethics Committee of the Affiliated Hospital of Jiangnan University (No. LS2019027), and written informed consent was obtained from all patients. All the patients were followed-up over telephone up to October 31, 2017, in order to analyze patient survival. The median follow-up was 80 months (range; 8–115 months).

### Tissue Microarray Construction and Immunohistochemistry Staining

Two experienced pathologists examined the section stained with hematoxylin and eosin (HE) and marked the carcinoma sites in the corresponding paraffin block. The detailed tissue microarray (TMA) construction protocol followed was as described previously (18). Briefly, the TMAs were constructed by obtaining a tissue cylinder of 1.0-mm in diameter core tissue biopsies from representative paraffin-embedded sections of each donor tissue block and implanted into the hole of the premade recipient paraffin block. The tissue paraffin blocks were serially sectioned into 4 μm thick sections, deparaffinized in xylene, and hydrated in an ethanol gradient. Antigen retrieval was performed by heating the tissue sections at 100°C in sodium citrate buffer for 30 min. Moreover, endogenous peroxidase was blocked by incubation in 3% hydrogen peroxide for 10 min. Subsequently, the sections were incubated in 5% bovine serum albumin (BSA) at room temperature (RT) for 30 min. Primary antibody, mouse anti-human B7-H3 monoclonal antibody (1:200, Santa Cruz, CA, United States) added dropwise followed by overnight incubation at 4°C. Following incubation, the slides were washed and incubated with a horseradish peroxidase-conjugated secondary antibody for 30 min. The immunostaining was carried out by staining with 3, 3’-diaminobenzidine chromogen (DAB) (GTVisionII Immunohistochemistry Detection Kit for Rabbit/Mouse, GeneTech, China) and counter-stained with hematoxylin, dehydrated, and mounted and the sections were examined under a microscope. Two independent pathologists evaluated the percentage of positive cells (score of 0: ≤5%; score of 1: 6–25%; score of 2: 26–50%; score of 3: 51–75%; score of 4: >76%) and the intensity [0 for negative staining (no coloration); 1 for weakly positive (faint yellow); 2 for moderately positive (yellowish brown); 3 for strongly positive (brown)] of all samples. The two scores were multiplied to generate the final score for each specimen range from 0 to 12.

### Verification of the Cut-Off Value

We analyzed the receiver operating characteristics (ROC) curve of B7-H3, FBG, LDH, and FMN through SPSS 23.0 software (IBM, United States). Then, the Youden Index was calculated according to the formula sensitivity + specificity – 1. The maximum Youden Index representing the expression value of the target was considered as the cut-off value. Based on values, patients with less than cut-off value was defined as low expression group and those with a value equal or greater than cut-off value as described as high expression group.

### Statistical Analysis

The relationship between clinicopathological parameters and B7-H3, FBG, LDH, or FMN was analyzed using the Chi-square test. The Mann–Whitney test was used to analyze the difference of the expression of B7-H3, FBG, LDH, and FMN for each clinical characteristic. The non-parametric Spearman test evaluated the correlation of B7-H3 and FBG, LDH, or FMN expression in CRC patients. Overall survival (OS) was plotted using the Kaplan–Meier method and compared using the log-rank test. The Cox proportional hazard model was used to perform multivariate survival analysis. The R software version 3.6.1 and the RMS package (R Foundation for Statistical Computing) were used to perform the nomogram analysis and calibration plot. All data were analyzed using the SPSS package (version 23.0, IBM, Chicago, IL, United States). All figures were generated with GraphPad Prism 6.0 (GraphPad Software Inc., United States). A *P*-value of <0.05 was considered statistically significant.

## Results

### Clinicopathological Parameters of Patients

The clinicopathological parameters of 213 CRC patients were summarized in Table 1. The study comprised 118 males (55.4%) and 95 females (44.6%). The age at initial diagnosis was classified into <60 years (37.6%) or ≥60 years (62.4%), and the median age of the patients was 62 (range, 27–87 years old). 38.5% of tumors were located or distributed in the colon (*n* = 82) and 61.5% of tumors were located in the rectum (*n* = 131). Lymph node metastasis (N1–N2) was noted in 43.7% of cases. There were 115 (54.0%) cases with stage I-II disease and 98 (46.0%) cases with stage III–IV disease.

**TABLE 1:** Association of B7-H3, FBG, LDH, and FMN expression in CRC patients with clinicopathological parameters.

Clinical parameter	Case (*n*)	B7-H3 expression		*p* value	FBG expression		*p* value	LDH expression		*p* value	FMN expression		*p* value
		Low	High		Low	High		Low	High		Low	High	
Gender				**0.028**			0.216			0.545			0.718
Male	118	35	83		75	43		78	40		92	26	
Female	95	42	53		68	27		59	36		76	19	
Age (years)				0.572			0.831			0.872			0.467
<60	80	27	53		53	27		52	28		61	19	
≥60	133	50	83		90	43		85	48		107	26	
Tumor location				0.098			0.237			**0.048**			0.252
Colon	82	24	58		59	23		46	36		68	14	
Rectum	131	53	78		84	47		91	40		100	31	
Colon cancer site				0.371			0.655			0.763			0.212
Left-sided	176	66	110		117	59		114	62		136	40	
Right-sided	37	11	26		26	11		23	14		32	5	
Depth of tumor invasion				0.130			0.335			0.231			0.588
T1/2	64	28	36		46	18		45	19		49	15	
T3/4	149	49	100		97	52		92	57		119	30	
Lymph node metastasis				0.494			0.192			0.600			**0.013**
N0	120	41	79		85	35		79	41		102	18	
N1/2	93	36	57		58	35		58	35		66	27	
Distant metastasis				0.428			0.968			**0.009**			0.063
Yes	15	4	11		10	5		5	10		9	6	
No	198	73	125		133	65		132	66		159	39	
TNM stage				0.870			0.161			0.384			**0.014**
I/II	115	41	74		82	33		77	38		98	17	
III/IV	98	36	62		61	37		60	38		70	28	
Neural invasion				0.685			0.288			0.479			0.917
Yes	39	13	26		29	10		27	12		31	8	
No	174	64	110		114	60		110	64		137	37	
Vascular invasion				0.307			0.554			0.337			0.278
Yes	35	10	25		25	10		25	10		30	5	
No	178	67	111		118	60		112	66		138	40	
Mucinous adenocarcinoma				0.707			0.831			0.124			0.897
Yes	20	8	12		13	7		16	4		16	4	
No	193	69	124		130	63		121	72		152	41	
Differentiation				0.461			0.538			0.739			0.284
Well	76	25	51		49	27		50	26		63	13	
Moderate/Poor	137	52	85		94	43		87	50		105	32	

*LDH, lactic dehydrogenase; FMN, fructosamine; FBG, fasting blood glucose; OS, overall survival. Bold values mean significant p value which is < 0.05.*

### Expression of B7-H3, FBG, LDH, and FMN in CRC Patients

**FIGURE 1:**
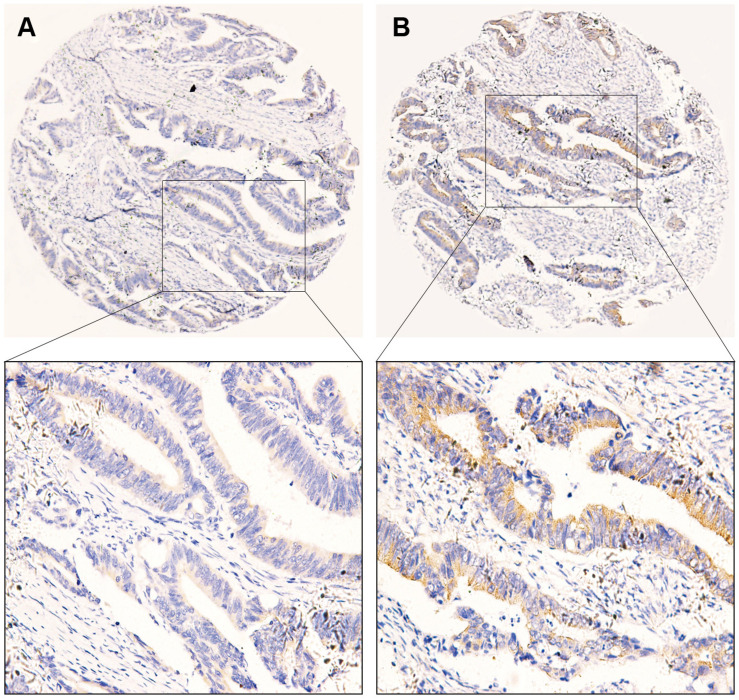
Representative images of immunohistochemical staining of B7-H3 expression in colorectal cancer tissue. **(A)** Low expression of B7-H3 (40×). **(B)** High expression of B7-H3 (40×). The images below show the magnification of each zone (200×). Scale bar = 50μm.

Using immunochemistry, we detected the expression of B7-H3 in CRC tissue. According to the cut-off value of 2.5, scores of 0–2 were defined as the low expression group, and scores of 3–12 were considered as the high expression group. Similar to previous studies, B7-H3 was highly expressed in CRC tissue. The positive rates of B7-H3 expression were 63.8% (136/213). Furthermore, positive staining of B7-H3 was predominantly localized in the cytoplasm and membrane of tumor cells. The representative images were presented in Figure 1. Similarly, based on the value of FBG, LDH, and FMN from the clinical laboratory and survival outcome of patients, we calculated the cut-off value using the Youden Index. Thus, patients were defined as either low expression of FBG with the value <5.425 or high expression of FBG with the value ≥5.425. We found that 32.9% of cases exhibited high expression of FBG (70/213). Also, the cut-off value of LDH and FMN were defined as 169.5 and 202.5, respectively, and the corresponding positive rates of LDH and FMN expression in patients with CRC were 35.7% (76/213) and 21.1% (45/213).

### Correlation Between B7-H3, FBG, LDH, and FMN and Clinicopathological Characteristics of Patients With CRC

**FIGURE 2:**
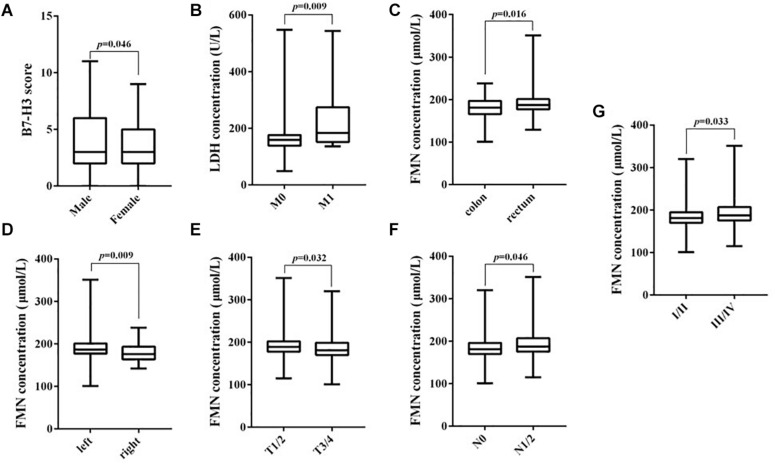
Correlation between B7-H3, LDH, and FMN expression and clinicopathological parameters. **(A)** B7-H3 and gender; **(B)** LDH and distant metastasis; **(C)** FMN and tumor location; **(D)** FMN and colon cancer site; **(E)** FMN and depth of tumor invasion; **(F)** FMN and lymph node metastasis; **(G)** FMN and TNM stage.

A significant difference between the expression of B7-H3 and the gender of patients with CRC was found (*p* = 0.028). Notably, positive expression of LDH was significantly associated with tumor location (*p* = 0.048) and distant metastasis (*p* = 0.009). Furthermore, the FMN expression was significantly related to the lymph node metastasis (*p* = 0.013) and the TNM stage (*p* = 0.014). However, the expression of FBG exhibited no significant association with any clinicopathological characteristics (Table 1). Using the Mann–Whitney test, we confirmed the marked differences between B7-H3 and gender (*p* = 0.046), LDH, and distant metastasis (*p* = 0.009). Notably, the expression of FMN was significantly associated with tumor location (*p* = 0.016), colon cancer site (*p* = 0.009), depth of tumor invasion (*p* = 0.032), lymph node metastasis (*p* = 0.046), and TNM stage (*p* = 0.033) (Figure 2).

### Correlation Between B7-H3 and FBG, LDH, or FMN Expression in CRC

**FIGURE 3:**
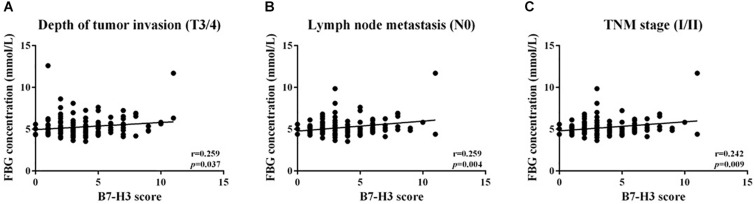
Positive linear relationship between B7-H3 and FBG. **(A)** Depth of tumor invasion (T3/4); **(B)** lymph node metastasis (N0); **(C)** TNM stage (I/II).

To determine whether B7-H3 expression associated with FBG, LDH, or FMN expression in CRC, correlations analysis was conducted. However, there was no significant linear relationship between the expression of B7-H3 and FBG, LDH or FMN in all CRC tissue. Further, we analyzed the correlation between B7-H3 and FBG, LDH or FMN in different stages. We found a linear correlation between B7-H3 and FBG with depth of tumor invasion (T3/4) (*p* = 0.037, *r* = 0.259), lymph node metastasis (N0) (*p* = 0.004, *r* = 0.259) and TNM stage (I/II) (*p* = 0.009, *r* = 0.242) (Figure 3). These findings indicated a significant correlation between the expression of B7-H3 and FBG in the early stage of CRC.

### Prognostic Value of B7-H3, FBG, LDH, and FMN Expression

**FIGURE 4:**
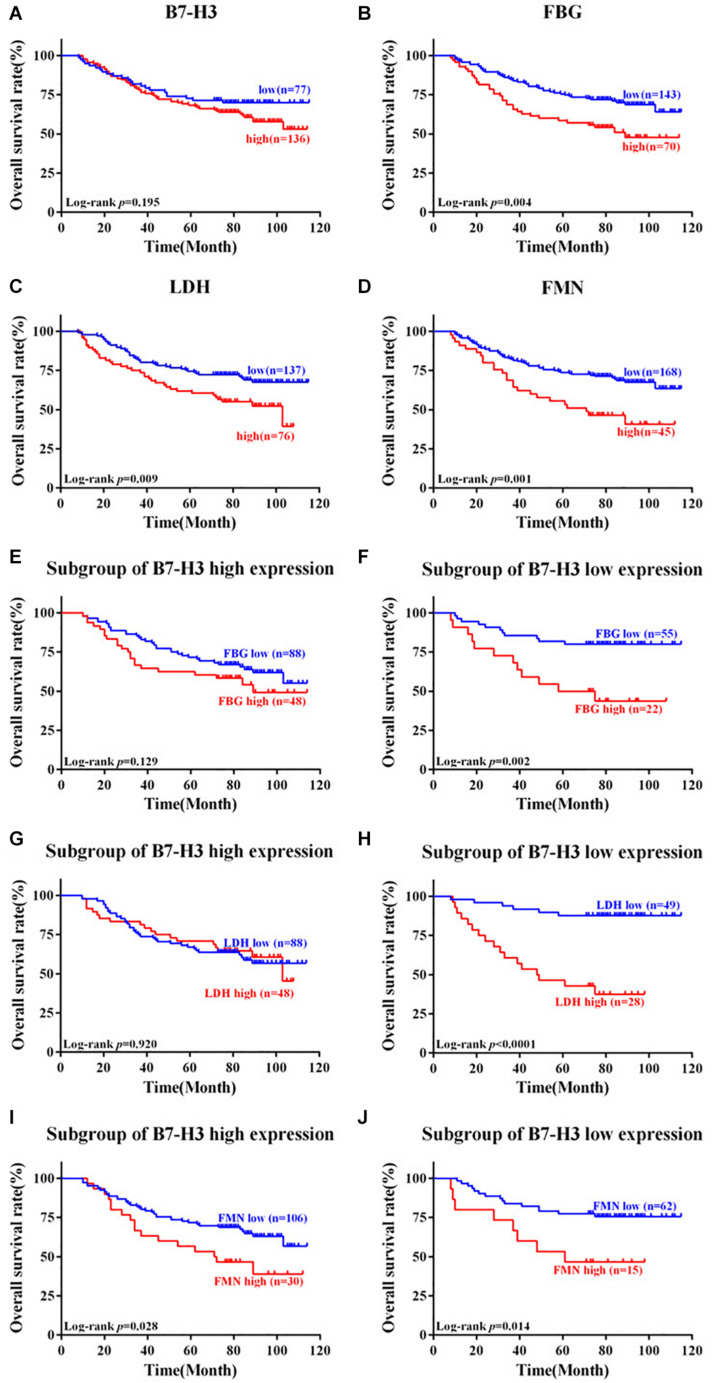
Kaplan-Meier survival curves of patients with colorectal cancer expressing B7-H3, FBG, LDH, and FMN. **(A)** Patients with low expression of B7-H3 vs. high expression of B7-H3. **(B)** Patients with low expression of FBG vs. high expression of FBG. **(C)** Patients with low expression of LDH vs. high expression of LDH. **(D)** Patients with low expression of FMN vs. high expression of FMN. **(E)** Subgroup of patients with high expression of B7-H3 and low expression of FBG vs. high expression of FBG. **(F)** Subgroup of patients with low expression of B7-H3 and low expression of FBG vs. high expression of FBG. **(G)** Subgroup of patients with high expression of B7-H3 and low expression of LDH vs. high expression of LDH. **(H)** Subgroup of patients with low expression of B7-H3 and low expression of LDH vs. high expression of LDH. **(I)** Subgroup of patients with high expression of B7-H3 and low expression of FMN vs. high expression of FMN. **(J)** Subgroup of patients with low expression of B7-H3 and low expression of FMN vs. high expression of FMN.

OS was computed from the date of surgery until the patient’s death. The 5-year survival rate was 63.4% (135/213) for patients with CRC in this study. We respectively analyzed the associations between OS and the expression of B7-H3, FBG, LDH, or FMN. Although the expression of B7-H3 did not affect the OS of patients (*p* = 0.195) (Figure 4A), notably, patients with the high expression of FBG, LDH, or FMN exhibited a significantly worse OS compared to patients with low expression (*p* = 0.004, *p* = 0.009, and *p* = 0.001) (Figures 4B–D). Furthermore, we also evaluated the correlation between OS and FBG, LDH, or FMN in the subgroup with different expression of B7-H3. Interestingly, in the subgroup with high B7-H3 expression, the only subgroup with high FMN expression exhibited significantly worse OS compared to the subgroup with high FBG or LDH expression (*p* = 0.028) (Figures 4E,G,I). However, a subgroup of patients with low expression of B7-H3 and high FBG, or high LDH, or high FMN exhibited significantly worse prognosis as compared to patients with low expression of FBG, LDH or FMN (*p* = 0.002, *p* < 0.0001, and *p* = 0.014) (Figures 4F,H,J).

Furthermore, to evaluate the risk factors associated with the prognosis of patients with CRC, univariate Cox proportional hazard model analysis was performed. As represented in Table 2, depth of tumor invasion (T3/4), lymph node metastasis (N1/2), distant metastasis, TNM stage (III/IV), neural invasion, high expression of FBG, high expression of LDH, high expression of FMN, high expression of both B7-H3 and FMN, low expression of B7-H3 and high expression of FBG, low expression of B7-H3 and high expression of LDH, low expression of B7-H3 and high expression of FMN were highly correlated with the OS of patients with CRC. Besides, the multivariate analysis revealed that distant metastasis and high expression of FBG were significant independent prognostic factors for OS of patients with CRC (Table 2).

**TABLE 2:** Univariate and multivariate analyses of clinical parameters associated with OS in CRC patients.

Clinical parameter	Univariate analysis		Multivariate analysis	
	HR (95%CI)	*p* value	HR (95%CI)	*p* value
Gender (male vs. female)	0.876 (0.559–1.373)	0.563		
Age (≥60 vs. <60)	1.201 (0.753–1.916)	0.441		
Tumor location (colon vs. rectum)	0.770 (0.492–1.206)	0.254		
Colon cancer site (right-sided vs. left-sided)	1.112 (0.624–1.983)	0.719		
Depth of tumor invasion (T3/4 vs. T1/2)	2.481 (1.367–4.503)	**0.003**	1.812 (0.972–3.372)	0.061
Lymph node metastasis (N1/2 vs. N0)	2.445 (1.547–3.865)	**0.000**	0.597(0.177–2.015)	0.406
Distant metastasis (yes vs. no)	11.646 (6.364–21.312)	**0.000**	5.949 (2.788–12.697)	**0.000**
TNM stage (III/IV vs. I/II)	3.001 (1.871–4.814)	**0.000**	3.349 (0.911–12.307)	0.069
Neural invasion (yes vs. no)	1.726 (1.037–2.871)	**0.036**	1.219 (0.699–2.126)	0.484
Vascular invasion (yes vs. no)	1.565 (0.914–2.681)	0.103		
Mucinous adenocarcinoma (no vs. yes)	0.792 (0.344–1.824)	0.584		
Differentiation (well vs. moderate/poor)	0.786 (0.500–1.236)	0.297		
B7-H3 (high vs. low)	1.377 (0.846–2.240)	0.198		
FBG (high vs. low)	1.916 (1.223–3.000)	**0.005**	1.733 (1.050–2.861)	**0.032**
LDH (high vs. low)	1.801 (1.153–2.813)	**0.010**	1.430 (0.902–2.266)	0.128
FMN (high vs. low)	2.154 (1.336–3.472)	**0.002**	1.377 (0.807–2.352)	0.241
B7-H3 high (FBG high vs. low)	1.444 (0.841–2.479)	0.183		
B7-H3 high (LDH high vs. low)	0.972 (0.557–1.695)	0.920		
B7-H3 high (FMN high vs. low)	1.881 (1.059–3.339)	**0.031**		
B7-H3 low (FBG high vs. low)	3.393 (1.493–7.709)	**0.004**		
B7-H3 low (LDH high vs. low)	7.107 (2.785–18.138)	**0.000**		
B7-H3 low (FMN high vs. low)	2.800 (1.184–6.625)	**0.019**		

*Bold values mean significant p value which is < 0.05.*

### Development and Validation of Nomograms for Predicting Prognosis

**FIGURE 5:**
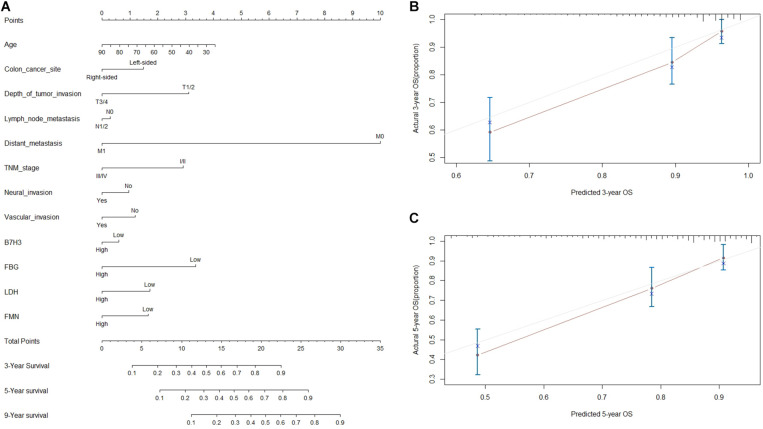
Nomogram based prediction of prognosis. **(A)** Nomogram for OS based on B7-H3, FBG, LDH, FMN, and other clinicopathological characteristics. **(B)** The calibration of the nomogram for OS of 3 years. **(C)** The calibration of nomogram for OS of 5-year.

To envisage the prognostic significance of B7-H3, FBG, LDH, and FMN expression, we generated nomograms for OS based on the expression of B7-H3, FBG, LDH, and FMN, and other clinicopathological characteristics (Figure 5A). Using nomogram construction, we could identify the score on the point-scale corresponding to significant factors of each subtype. Then, the total points of each patient were calculated by adding up the score of independent variables altogether. Lastly, by analyzing the complete point scale, we were able to estimate the probability of survival at different time points. The findings indicated that distant metastasis was a significant determinant of prognosis, followed by the FBG expression, depth of tumor invasion, and TNM stage. Overall, it implied that, the larger the score, the higher the survival rate, and *vice versa*. Subsequently, we were able to predict the prognosis based on nomogram analysis. Figures 5B,C showed the 3-year and 5-year calibration of the nomogram for OS, establishing a pronounced prediction accuracy of this nomogram, indicating that calibration curves for nomogram revealed no deviations from the reference line.

## Discussion

Colorectal cancer remains one of the most frequently diagnosed malignancies worldwide. Increasing pieces of evidence support that the majority of CRC are sporadic, which are predominantly attributable to the constellation of modifiable risk factors characterizing westernization as the significant risk factor (19). These risk factors have also been consistently related to the occurrence of DM (20). In addition, the risk of developing CRC was estimated to be 27% higher in patients with type 2 DM compared to non-diabetic controls (21). Recently, a meta-analysis by Zhu et al. suggested that diabetes had a negative effect on CRC in OS (22). DM was a chronic disease mainly characterized by disturbances in glucose metabolism (23). Besides, altered energy metabolism is one of the biochemical fingerprints of cancer cells, representing it as one of the hallmarks of cancer, including CRC. Colorectal cancer has also been characterized by altered glucose metabolism mediated by glycolytic pathways (24). Although conceptual advances have significantly improved the understanding on the clinical significance of tumor metabolism, only limited studies have been focused on B7-H3 as indicators of glucose metabolism in CRC patients. Therefore, this study investigated the survival outcome of patients with CRC by the indicators of glucose metabolism.

In the present study, three clinical indicators most frequently used in our hospital were selected, including FBG, LDH, and FMN. FBG was measured as the major criterion of DM, indicating the level of glucose in the blood. LDH is a key enzyme of glycolysis, which catalyzes the inter-conversion of pyruvate and lactate, and it also converts pyruvate, the final product of glycolysis, to lactate in the absence of oxygen. The high expression of LDH can reveal abnormal glucose metabolism. Fructosamine is formed through glycosylation of serum protein, such as albumin. The concentration of FMN in serum directly reflects blood glucose concentration precisely than HbA1c.

Furthermore, a deregulated or altered energy metabolism has been predominantly recognized as the “hallmarks of cancer.” Recently, studies have implicated different roles of lactate export/import contributing to the survival and growth of cancer. In this study, the expression of LDH was significantly correlated with tumor location and distant metastasis, the expression of FMN was notably associated with lymph node metastasis and TNM stage; however, no significant relationship between the expression of FBG and clinicopathological characteristics were recorded. Next, a significant correlation between LDH expression and distant metastasis, also between FMN expression and tumor location, colon cancer site, depth of tumor invasion, lymph node metastasis, and TNM stage was demonstrated with Mann Whitney test. In contrast, FBG expression had no relation with pathological features. These findings suggested the respective expression of LDH or FMN had a closer relationship with the parameters of CRC patients than FBG expression in this study. Moreover, high expression of FBG, LDH, or FMN all indicated poor OS of CRC patients eloquently. Conceivably, FBG, LDH, and FMN could predict the prognosis of patients with CRC as an independent prognostic factor. Clinically, the value of FBG, LDH, and FMN could be controlled to perceive the progression in patients with CRC.

Treatment with immune checkpoint inhibitors has emerged as a frontline treatment for patients with CRC, mainly targeting cytotoxic T-lymphocyte antigen 4 (CTLA-4) and programed death-1 receptor (PD1) and its ligand PD-L1 (25). B7-H3, as one of the crucial immune checkpoint proteins, plays a critical role in the occurrence and development of CRC. Clinically, the expression of B7-H3 was associated with unfavorable outcomes in CRC patients (12, 26). However, there was no relationship between B7-H3 expression and OS in this study, which might be attributed to low sample size or the uncertainty of repeated experiments. Nevertheless, over 1000 cases were included in other investigations in order to predict the prognosis of patients. It is noteworthy that the overexpression of B7-H3 has been suggested as a predictor of poor OS in our previous study with more than 200 patients. Thus, the prognostic significance of B7-H3 warrants further validation in future investigation. Therefore, B7-H3 was still a feasible and effective marker to predict the prognosis in patients with CRC. Besides, recently, B7-H3 antibodies had been reported to be safely used in humans in early phase clinical trials (27, 28). Therefore, B7-H3 might serve as a reliable biomarker and therapeutic target for CRC.

Previously, our team has analyzed the non-immunological role of B7-H3 in CRC tumorigenesis, which mainly focused on anti-apoptosis (29), pro-metastasis (30, 31), 5-Fu resistance (32, 33), and lipid metabolism (34); however, the relationship between B7-H3 and glucose metabolism was not investigated. Recently, Shi et al. suggested that B7-H3 might be a novel regulator of glucose metabolism via regulating HK2 expression in CRC cells (14). It motivated us to explore the clinical and prognostic significance of B7-H3 expression as indicators of glucose metabolism in patients with CRC. Thus, we evaluated the clinical correlation and performed survival analysis in patients with CRC for the combined expression of B7-H3 with FBG, LDH, and FMN. We found a linear relationship between B7-H3 and FBG with the depth of tumor invasion (T3/4), lymph node metastasis (N0), and TNM stage (I/II). However, there were no significant linear relationships between the expression of B7-H3 and FBG, LDH, or FMN in all CRC tissue, and also between B7-H3 and LDH or FMN among different subgroups. These results indicated a positive correlation between the expression of B7-H3 and FBG in the early stage of CRC tissue. Furthermore, we considered analyzing the risk of CRC by investigating the aberrant expression of B7-H3 and FBG. In the future, we anticipate detecting B7-H3 and FBG in biopsy tissue specimens and serum of patients with CRC. However, it would possibly be beneficial if the expression of B7-H3 could also be determined in the serum of patients. Further, a subgroup of B7-H3 high expression revealed that only patients with high FMN expression exhibited significantly worse OS compared with FBG and LDH expression. However, a subgroup of patients with low expression of B7-H3 and high FBG, or high LDH, or high FMN exhibited significantly worse prognosis as compared to patients with low expression of FBG, LDH, or FMN. These shreds of evidence implied that FMN is a crucial factor for OS prediction in CRC patients irrespective of high or low expression of B7-H3. Besides, high expression FBG or LDH could warn poor survival outcomes when B7-H3 expression was low. Possibly, in the subgroup of patients with low expression of B7-H3, indicators of glucose metabolism (FBG, LDH, and FMN) should be monitored continually for improved evaluation of prognosis in these patients. Moreover, Cox regression analysis and nomogram manifested the contribution of different clinicopathological characteristics to OS. Conceivably, the survival status and rate of CRC patients could be estimated to stratify the patients for clinical treatment appropriately.

The present study revealed the clinical and prognostic significance of B7-H3 and the clinical indicators of glucose metabolism (FBG, LDH, and FMN) in patients with CRC. Collectively, the findings of this study presented B7-H3 as a hub molecular biomarker, which, combined with indicators like FBG, LDH, or FMN, could possibly reflect the clinical outcomes of patients with CRC. B7-H3 intervention and control of glucose metabolic level appeared to be a promising antitumor strategy with possible benefits in clinical translation. In conclusion, this study provided new insight into the relationship of B7-H3 and glucose metabolism in patients with CRC.

## Data Availability Statement

All datasets presented in this study are included in the article/supplementary material.

## Ethics Statement

The studies involving human participants were reviewed and approved by Medical Ethics Committee of the Affiliated Hospital of Jiangnan University. The patients/participants provided their written informed consent to participate in this study. Written informed consent was obtained from the individual(s) for the publication of any potentially identifiable images or data included in this article.

## Author Contributions

TZ, XQ, YM, and DH designed the research. TZ, YJ, XJ, and LL performed the research. TZ and LL analyzed the data. TZ, YJ, XJ, and LL wrote the manuscript. XQ, YM, and DH revised the manuscript. All authors contributed to the article and approved the submitted version.

## Conflict of Interest

The authors declare that the research was conducted in the absence of any commercial or financial relationships that could be construed as a potential conflict of interest.
